# Chronic Recurrent Multifocal Osteomyelitis Mimicking Scurvy in a Child: A Case Report

**DOI:** 10.7759/cureus.38880

**Published:** 2023-05-11

**Authors:** Rawan Alhalabi, Basil Nasrallah, Rizviya Rahman, Hussein Muad, Assad Qureshi

**Affiliations:** 1 Department of Pediatrics, American Hospital Dubai, Dubai, ARE; 2 Department of Orthopaedic Surgery, American Hospital Dubai, Dubai, ARE

**Keywords:** child nutrition, persistent leg pain, leg swelling, joint pain, chronic recurrent multifocal osteomyelitis (crmo), vitamin c deficiency, scurvy

## Abstract

Scurvy is a rare clinical syndrome resulting from prolonged vitamin C deficiency and is uncommon in the Gulf area. It can present with non-specific symptoms, making diagnosis and treatment challenging. In pediatric patients, symptoms may include weight loss, lethargy, low-grade fever, anemia of varying degrees, easy bruising or bleeding, joint and muscle pain, and poor wound healing. Despite advances in healthcare in many Gulf countries, nutritional deficiencies can still occur in certain populations. Therefore, it is important for pediatricians, orthopedists, rheumatologists, and radiologists to consider scurvy in the evaluation of children with low-grade, multisystemic involvement. We report a case of a six-year-old boy who presented to the emergency department (ED) multiple times with progressive right (RT) leg pain. The clinical picture and imaging findings suggested chronic recurrent multifocal osteomyelitis (CRMO). Despite symptom progression, scurvy was ultimately diagnosed and treatment with vitamin C led to rapid resolution of his symptoms. This case highlights the importance of considering scurvy in the differential diagnosis of children with multisystemic involvement, especially in regions where nutritional deficiencies may be more prevalent.

## Introduction

Vitamin C deficiency, clinically manifested as scurvy, is the clinical syndrome of prolonged ascorbic acid deficiency. Hippocrates (460 BC) initially described it as follows: "the gums separate from the teeth, blood runs from the nostrils, black-colored ulcerations frequently appear on the legs, some heal, others do not, and the skin is thin..." [1]. Although historically observed in the era of great maritime expeditions due to poor dietary intake, it still occurs in the modern age in certain settings. A scurvy outbreak recently occurred in a refugee camp in Kenya in 2018 [1-3]. Scurvy is more common in impoverished and middle-income countries while being relatively uncommon in high-income settings [4]. Statistics from the National Health and Nutrition Examination Survey (NHANES) suggest that less than 2% of the population of the USA aged 6-11 years are vitamin C deficient, and women had higher mean concentrations of vitamin C than did men [5].

Though vitamin C deficiency is well understood, overt scurvy is rare. The most frequent latent scurvy symptoms in infants and young children are non-specific, such as lethargy, irritability, vague pain in the muscles or joints of the lower limb, difficulty walking, weight loss, and bleeding gums. Other manifestations include loss of appetite, low-grade fever, anemia of a variable degree, tendency to bruise or bleed easily at any site, petechial hemorrhages, poor wound healing, and dry rough skin [6-8]. Numerous risk factors that contribute to scurvy have been identified, predominantly relating to malnutrition, including eating disorders, drug and alcohol dependence, inflammatory bowel disease, and socioeconomic deprivation [1,3,9-13].

The varied presentation of vitamin C deficiency gives rise to many differentials depending on the pattern of symptoms and clinical signs. A bleeding tendency may be attributed to a hematological cause, such as Henoch-Schonlein purpura, disseminated intravascular coagulation, immune thrombocytopenic purpura, deep venous thrombosis, or vasculitis, among many others. Lesions within muscle and bone due to the propensity to bleed can be wrongly mislabeled as acute lymphoblastic leukemia, Ewing's sarcoma, or osteomyelitis [7].

Chronic recurrent multifocal osteomyelitis (CRMO) is defined by the American College of Rheumatology as a disorder causing bone pain due to inflammation in the absence of detectable infection. There may be an accompanying impairment in weight-bearing or function and occasionally joint swelling. Although inflammatory markers may be raised, they can be normal, which makes the diagnosis of CRMO elusive. Therefore, CRMO is often viewed as a diagnosis of exclusion. Many tests are often required, including bone scan, MRI, and occasionally bone biopsy. The condition responds to non-steroidal anti-inflammatory drugs (NSAIDs), but some patients may require immunosuppressive drugs such as methotrexate [14].

The wide-ranging symptoms of scurvy coupled with the differential diagnosis of CRMO, which can be elusive to detect, can introduce significant delays in correctly identifying the underlying vitamin C deficiency and instituting treatment. We report a case of a six-year-old boy who presented to the emergency department (ED) complaining of progressive right (RT) leg pain after minor trauma. He was incorrectly diagnosed with CRMO after excluding infectious osteomyelitis, hematological disorders, malignancies, and autoimmune disorders. Eventual suspicion of a vitamin C deficiency prompted an assessment of levels, confirming the deficiency as the underlying cause. The child improved substantially after receiving the suitable ascorbic acid dose.

## Case presentation

A previously healthy six-year-old Arabic boy from an unremarkable socioeconomic background sustained minor trauma to his left leg after falling at school a few days prior to his first visit to the ED (day 1). He initially complained of left leg pain around the knee joint, which improved spontaneously over the following days. The child presented again on day 7 with persistent RT leg pain despite a course of analgesia, including NSAIDs. The patient developed a limp and eventually refused to weight-bear on the right side on day 10. The mother noticed mild RT knee swelling, which developed a few days after the initial trauma. The patient was also noted to have blood collection in his gum and bilateral lower limb petechiae. There was no weight loss, fever, anorexia, night sweats, or skin bruises. On physical examination, he was afebrile, alert, and active. A hematoma was noted in his gum. A mild petechial rash with a non-dermatomal distribution was detected. There was swelling of the RT knee with warmth and mild restriction in the range of movement. He was unable to fully weight-bear on the right side and had a demonstrable limp. His vital signs were unremarkable. He was admitted for further assessment as osteomyelitis was suspected. Laboratory investigations were within normal ranges, including C-reactive protein (CRP), creatine kinase (CK), and urine analysis. X-rays of the hips were unremarkable on the seventh day of his first presentation.

**Figure 1:**
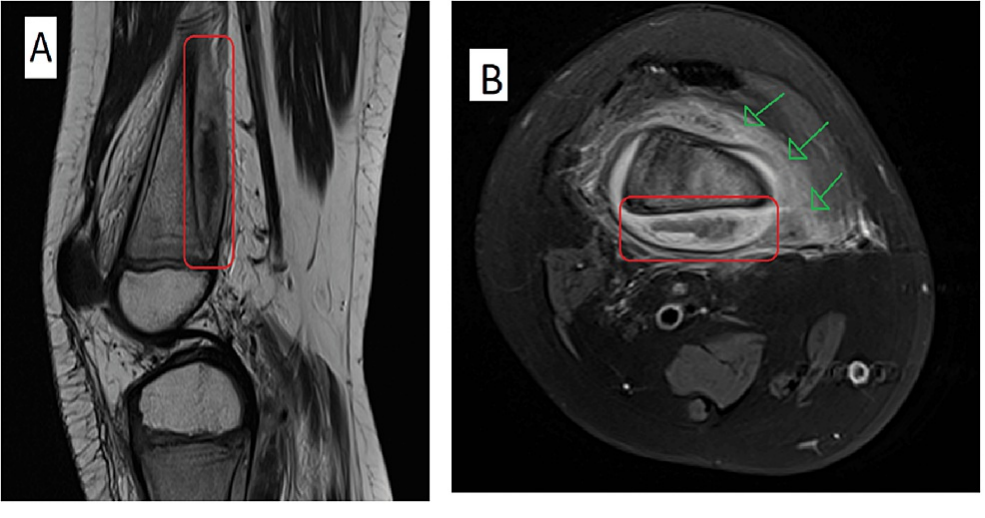
MRI RT knee. (A, B) Distal femur subperiosteal collection (red rectangular). (B) Femur surrounding soft tissue edema (green arrows). RT: right.

During his hospital stay, his CRP was rechecked and found to be elevated (43, ref. range: ≤5 mg/dL) with a normal white blood cell count and hemoglobin level. Radiographs of the RT knee revealed a soft tissue collection around the distal femur with no evidence of cortical bone destruction. On day 11, subsequent MRI demonstrated a diffuse periosteal reaction involving the distal femur and proximal tibia with surrounding soft tissue edema and suspected subperiosteal collection (Figure 1).

Based on the imaging findings and blood tests, a presumptive diagnosis of CRMO was made pending further assessment by bone biopsy. The biopsy procedure, on day 12, identified an obvious hematoma overlying the posterior aspect of the distal femur with no evidence of infection. Muscle tissue overlying the periosteum, a small piece of the periosteum, and bone marrow were sampled for culture, sensitivity, and histopathology. A blood sample was aspirated from the bone marrow for cytology and histopathology. The patient was clinically stable postoperatively and intravenous antibiotics were administered. Histology, culture, and sensitivity all failed to return any evidence of infection or a tissue diagnosis of malignancy. The results of further blood tests conducted on day 15 are shown in Table 1.

**Table 1:** Blood test results on day 15. CRP: C-reactive protein.

Test	Result	Reference range
WBC	2.8 ↓	5–15 10^9 ^\L
RBC	3.34 ↓	4–5.2 10^12 ^\L
Hemoglobin	85 ↓	115–155 g\L
Vitamin D 25-hydroxy	31 ↓	75–250 nmol\L
CRP	39.4 ↑	0–5 mg\L
Ferritin	190 ↑	7–140 ug\L

A subsequent three-phase bone scan of both knees with single-photon emission computed tomography (SPECT)\CT of the whole body excluded any potential malignancy as the cause of the current clinical picture. However, despite 17 days passing since his initial visit, the leg pain, petechiae, gum hematoma, and limping persisted. Nutritional deficiency had not been considered until this point due to the lack of concern regarding the socioeconomic background of the patient. Although the family did not feel he had any nutritional issues, they admitted that he only consumes dairy products, including cheese and milk along with biscuits, with a profound absence of fruits or vegetables on direct questioning. The serum ascorbic acid level was measured and found to be critically low, <0.1 mg/dL (ref. value 0.4-2). The child was immediately started on vitamin C supplements and demonstrated a dramatic improvement in all symptoms within a short period of time. The petechiae disappeared within 28 hours; he started to walk after 72 hours and was able to bear weight after five days of ascorbic acid ingestion. On day 23 of his first ED visit, the child was discharged with almost full recovery of all his symptoms.

During one year of follow-up visits to the clinic, none of his complaints recurred. It should be noted that the child is taking vitamin C supplements daily while maintaining an exclusively dairy diet, including cheese and milk along with biscuits. Further investigations were unfavorable as the parents wanted to avoid more radiation exposure and psychological trauma to their child.

## Discussion

Vitamin C is an essential nutrient obtained solely from dietary intake. Although it is found in most fruits and vegetables, it is absent in specific food groups, rendering certain focused diets susceptible to inducing deficiency. Another factor that affects bioavailability following dietary intake is the thermal sensitivity of ascorbic acid, which leads to reduced nutritional value from boiling or cooking [11,15]. Vitamin C is a vital cofactor in collagen biosynthesis; it modifies the collagen into its effective structure by hydroxylating the proline and glycine amino acids [9,16]. Bleeding in scurvy results from vascular fragility due to compromised collagen modification. Vitamin C also plays an important role in the biosynthesis of carnitine, histamine, and several adrenal steroids. It promotes iron absorption and mobilization, activates the enzyme that converts dopamine to norepinephrine, and functions in tyrosine, folate, and xenobiotic metabolism [5]. Therefore, a persistent decrease in its serum concentration induces widespread effects in many different organs. Our case report emphasizes the various clinical manifestations of scurvy induced exclusively due to poor dairy intake. The presence of mucocutaneous changes, such as petechiae and gingival hematoma, can be elusive unless specifically sought. Other non-specific manifestations, such as lethargy, fever, and anorexia, make the diagnosis difficult [1,13]. Musculoskeletal effects in scurvy relate to an impairment in bone collagen formation. These manifestations often appear in the later stages of ascorbic acid deprivation and account for 80% of presentations of scurvy and CRMO [1,11,13,14]. Although lower extremity pain and swelling, subperiosteal hemorrhagic lesions, and soft tissue edema are typical MRI findings in scurvy [17], they usually prompt more common diagnoses such as infection, malignancy, and CRMO, as in the current described case.

Highlighting how easy it is for the two diagnoses to be confused, chronic non-bacterial osteomyelitis (CNO), also known as CRMO, occurs primarily in children and adults with a median age of nine years (our patient is in the same age group). The usual clinical presentations, which were all present in our case, are signs of bone inflammation, including localized skin redness, warmth, swelling, and bone pain. The metaphysis of long bones, such as the femur (which we encountered), tibia, or humerus, is commonly affected (74%) [18,19]. However, CRMO does not present with poor wound healing, dry rough skin, petechiae, or gingival hematoma. 

Although elusive to diagnose due to its relative infrequency in middle- to high-income populations, confirmation of diagnosis is readily achieved with the ease of effective simple treatment with ascorbic acid supplementation. Correctly attributing non-specific manifestations to ascorbic acid deficiency is easily validated by observing the reversal of symptoms and MRI changes shortly after commencing treatment. This case highlights that the clinician to be cognizant of this diagnosis and consider diagnostic testing of serum ascorbic acid levels in cases where this diagnosis is suspected. In this case, an earlier diagnosis would have obviated the need for several investigations, including an invasive tissue biopsy. In contrast to the ease of diagnosis confirmation, often a multitude of clinicians may review the patient before a specific diagnosis is sought. In the current case, clinical review was undertaken at various points of care by pediatrics, orthopedics, rheumatology, and radiology. This case carries an important learning point for all medical professionals who may be involved in the care of pediatric patients with musculoskeletal pain and varied systemic symptoms suggestive of ascorbic acid deficiency.

## Conclusions

Scurvy is a nutritional deficiency that can be easily prevented by consuming a balanced diet that includes fruits and vegetables. However, in certain at-risk groups, such as those with limited access to a variety of foods, scurvy can still occur. Healthcare providers need to have a high index of suspicion for scurvy in patients presenting with symptoms that can mimic other diseases, such as musculoskeletal pain resembling CRMO. A detailed clinical assessment, including a review of dietary habits and an oral examination, can help establish the diagnosis early and prevent unnecessary diagnostic testing and treatment.
